# SNHG1 Inhibits ox-LDL-Induced Inflammatory Response and Apoptosis of HUVECs *via* Up-Regulating GNAI2 and PCBP1

**DOI:** 10.3389/fphar.2020.00703

**Published:** 2020-05-27

**Authors:** Yuan Lu, Jue Xi, Yao Zhang, Wensu Chen, Fengyun Zhang, Chenzong Li, Zhirong Wang

**Affiliations:** ^1^Department of Cardiology, Affiliated Hospital of Xuzhou Medical University, Xuzhou, China; ^2^Department of Endocrinology, Affiliated Hospital of Xuzhou Medical University, Xuzhou, China

**Keywords:** small nucleolar RNA host gene 1, atherosclerosis, miR-556-5p, G protein subunit alpha i2, poly(rC) binding protein 1

## Abstract

Dysfunction of human endothelial cells is an important trigger for atherosclerosis. Oxidative low-density lipoprotein (ox-LDL) usually was used to stimulate the dysfunction of human umbilical vein endothelial cells (HUVECs). LncRNA SNHG1 (small nucleolar RNA host gene 1) is a cerebral infarction-associated gene. The present study was designed to investigate the role of SNHG1 in ox-LDL-induced HUVECs. Cell viability was evaluated by CCK-8 and MTT assay. Cell apoptosis was detected by flow cytometry analysis. Cell inflammatory response was evaluated by detecting LDH, IL-6, IL-1*β* levels. The results revealed that up-regulation of SNHG1 attenuated ox-LDL-induced cell injury and inflammatory response in HUVECs. Next, mechanism assays including RNA immunoprecipitation (RIP) assay, luciferase reporter assay, and RNA pull-down assay, helped us to identify the interaction between miR-556-5 and SNHG1. GNAI2 (G protein subunit alpha i2) and PCBP1 (poly(rC) binding protein 1) were identified as the downstream targets of miR-556-5p. SNHG1 regulated dysfunctions of ox-LDL-induced HUVECs *via* sponging miR-556-5p and up-regulating GNAI2 and PCBP1. SNHG1 attenuated cell injury and inflammatory response in ox-LDL-induced HUVECs via up-regulating both GNAI2 and PCBP1 at a miR-556-5p dependent way.

## Introduction

Endothelial cell (EC) dysfunction is regarded as an important marker for atherosclerosis at an early stage. Persistent adverse stimulations could lead to EC injury and apoptosis in atherosclerosis (Akhtar et al., 2015). Oxidative low-density lipoprotein (ox-LDL) can induce hyperlipemia and stimulate endothelial dysfunction and apoptosis *via* enhancing oxidative stress in ECs (Pandey et al., 2014). Inflammatory response induced by ox-LDL is verified as a key event in atherosclerosis though how ox-LDL induces EC injury is unknown, (Back et al., 2019). Therefore, it is of great significance to explore the downstream molecular mechanism of ox-LDL-induced EC injury.

Long noncoding RNAs (lncRNAs) are a group of noncoding RNAs (ncRNAs) with over 200 nt in length and have been studied over the past decade. The functions of lncRNA in HUVECs have been elucidated and revealed. For example, down-regulation of TUG1 alleviates ox-LDL-induced injury in HUVECs *via* modulation of the miR-148b/IGF2 axis (Wu et al., 2020). Knockdown of BZRAP1-AS1 suppresses HUVEC cell proliferation, migration, and angiogenesis in hepatocellular carcinoma (Wang et al., 2019). LncRNA XXYLT1-AS2 regulates HUVEC cell proliferation and adhesion by targeting FUS (Wang et al., 2020). Recently, lncRNAs are widely reported to function as the competing endogenous RNA (ceRNA) (Quan et al., 2019; Yan et al., 2019; Zhao et al., 2019). In competing endogenous RNA (ceRNA) mechanism, lncRNA serves as a sponge to competitively bind with miRNA and represses the inhibition of miRNA on the target mRNA (Tay et al., 2014). For instance, depletion of lncRNA XIST prevents renal interstitial fibrosis in diabetic nephropathy through sponging miR-93-5p to regulate CDKN1A (Yang et al., 2019). LncRNA SNHG16 acts as miR-205 sponge to modulate Smad2, thus regulating human aortic smooth muscle cell proliferation and migration (Lin et al., 2019). LncRNA MALAT1 inhibits hypoxia/reoxygenation-induced HUVEC cell injury *via* targeting the miR-320a/RAC1 axis (Zhu et al., 2020).

Small nucleolar RNA host gene 1 (SNHG1) has a neuro-protective effect mediated by HIF-1*α*/VEGF signaling in ischemic stroke (Zhang et al., 2018). SNHG1 acts as an endogenous sponge for miR-195 and elevates BCL2-Like Protein 2 expression to alleviate cardiomyocyte apoptosis (Zhang et al., 2018). Additionally, SNHG1 has been reported to alleviate cerebral infraction through inhibiting apoptosis of neuronal cells (Chen et al., 2019). However, it is unclear whether SNHG1 exerted functions in HUVECs. Our present study focused on investigating the function and molecular mechanism of SNHG1 in ox-LDL injured HUVECs.

## Materials and Methods

### Cell Culture and Treatment

American Tissue Culture Collection (ATCC; Manassas, VA) commercially provided the human umbilical vein endothelial cells (HUVEC cells) which were cultured in DMEM medium (Gibco, Grand Island, NY) with 10% Fetal Bovine Serum (FBS) (Gibco) in humid air with 5% CO_2_ at 37°C. To establish the atherosclerosis cell model, ox-LDL (20 μg/ml, 50 μg/ml, 100 μg/ml) procured from Solarbio (Beijing, China) was utilized to treat the HUVECs within the prescribed time limit. Besides, the cells treated without ox-LDL served as the control. The cultured cells were harvested at passages of 4–6 and used for further analyses.

### Cell Transfection

The extracted total RNA was acquired and reverse-transcribed into cDNA. To produce SNHG1 overexpression vector, the full-length sequence of SNHG1 was subjected to PCR amplification and then inserted to the pcDNA3.1 vector at EcoRI and XhoI sites, and the vacant vector served as the endogenous control. All plasmids and corresponding control used in this study, including sh-GNAI2, sh-NC, miR-556-5p-mimic, and NC-mimic were synthesized and produced by GenePharma (Shanghai, China). Lipofectamine 3000 (Invitrogen, Carlsbad, CA) was utilized for transfection with plasmids at the concentration of 30 nM.

### RNA Extraction and qRT-PCR Analysis

In accordance with the protocols of suppliers, Trizol reagent (Takara, Tokyo, Japan) was utilized to separate the total RNA of the cells. NanoDrop ND-1000 spectrophotometer (Thermo Fisher Scientific, Inc., Waltham, MA) was used to detect the concentration and quality of the RNA. The first strand cDNA was composed from the total RNA by the use of the PrimeScript RT reagent Kit (Takara). The SYBR Premix Ex Taq (Takara) was applied to conduct the qRT-PCR experiment on CFX Connect Real-Time System (Bio-Rad, Hercules, CA). 2^−ΔΔCt^ was utilized to calculate the relative expression with GAPDH or U6 served as endogenous controls. Primers' sequences (designed according to MIQE guideline) were provided in **Supplementary Table 1**.

### MTT Assay

Ninety-six-well plates were utilized to seed the HUVEC cells which were revealed to the ox-LDL at the concentration of 50 μg/ml for 12, 24, and 48 h. After that, the new medium which included the 0.5% MTT solution (Sigma-Aldrich, Darmstadt, Germany) was utilized to displace the previous medium which contained the ox-LDL. At 4 h post cultivation, DMSO solutions (Sigma-Aldrich) were supplemented to each well after removing the medium. A microplate reader was used to detect the absorbance at 490 nm after the formazan was dissolved. Under the treatment with 50 μg/ml ox-LDL for 24 h, the viability of HUVEC cells with diverse transfections were detected *via* MTT assay according to the protocol.

### CCK8 Assay

96-well plates were used to seed the cells treated with different doses of ox-LDL (at 20, 50 or 100 μg/ml) at 37°C for 24 h. Then each well of the plates was supplemented with the CCK8 solution (Dojindo Laboratories, Kumamoto, Japan) for cultivating at least 4 h. The microplate reader (Bio-Rad) was utilized to estimate the absorbance at 450 nm. Besides, CCK-8 assay was also employed to detect the viability of HUVEC cells under diverse transfections when treated with 50 μg/ml ox-LDL for 24 h.

### Flow Cytometry

Cells apoptosis was measured by transporting phosphatidylserine with an FITC Annexin V apoptosis kit (BD Biosciences, San Jose, CA). Briefly, HUVEC cells were treated with 50 μg/ml of ox-LDL for 24 h, and then processed to propidium and Annexin V-FITC in dark room for 10 min after being rinsed two times. Cell apoptosis was estimated by the utilization of Flow Cytometer (BD Biosciences).

### LDH Release and ELISA Assays

LDH Cytotoxicity Assay Kit (Sigma-Aldrich) was utilized to evaluate the LDH's activity in impaired cells. 96-well plates were used to seed the HUVEC cells at the density of 1 × 10^4^ per well under treatment of 50 μg/ml of ox-LDL for 24 h. On the basis of the suppliers' protocols, the kit was used to detect the LDH activity after disclosure of ox-LDL for 24 h. For the sake of testing inflammatory cytokines secretion, Human High Sensitivity ELISA Kit (Boster Bio, Pleasanton, CA) was utilized to analyze the collected cell mediums for IL-6 and IL-1*β*. In the end, the absorbance at 405 nm was detected, and the standard curves were used to count it.

### Luciferase Reporter Assay and RIP

The HUVEC cells underwent 24 h of treatment with 50 μg/ml of ox-LDL that was harvested for luciferase assay and RIP assay. In order to produce the SNHG1-WT, vectors of pmirGLO luciferase reporter (Promega, Madison, WI) were utilized to insert the sequence of SNHG1 flanking the complementary sequence encompassing miR-556-5p element. At the same time, SNHG1-Mut was acquired by the mutation of the sequences complementary to the seed region of miR-556-5p. Besides, the 3′UTR sequences of GNAI2 and PCBP1 as well as their mutated sequences without miR-556-5p binding sites were acquired and inserted to pmirGLO vectors for luciferase assays. After the cotransfection was completed, luciferase reporter system (Promega) was used to analyze the luciferase activity of indicated HUVEC cells. With regard to RIP assay, HUVEC cells were lysed in RIP lysis buffer containing proteinase inhibitors and RNase inhibitors. Magna RNA immunoprecipitation kit (Millipore, Bedford, MA) was applied to capture RNAs that could be precipitated by anti-Ago2 in different HUVEC cells. Then qRT-PCR was applied to estimate the enrichment of precipitated RNAs.

### Western Blot Analysis

Denaturing Sodium Dodecyl Sulfate-Polyacrylamide Gel Electrophoresis (SDS-PAGE) sample buffer was used to dissolve the cells by the standard method. Protein lysates of ox-LDL (50 μg/ml) induced HUVEC cells (for 24 h) were extracted using RIPA lysis buffer supplemented with cocktail of protease inhibitors (Roche Diagnostics, Indianapolis, IN) then were transferred onto nitrocellulose membranes after 10% SDS-PAGE was used to separate them. Then the Tris Buffered Saline (TBS) which contained 0.1% Triton X-100 and 5% nonfat milk was added into the membranes and blockaded at the temperature of 4°C after that. Overnight passed, the primary first antibodies, including anti-LDH (ab222910, 1/1,000; Abcam, Cambridge, MA), anti-IL-6 (ab6672, 1/1,000; Abcam), anti-IL-1β (AB10626, 1/1,000; Merck Millipore, Darmstadt, Germany), anti-GNAI2 (ab157204, 1/10,000; Abcam), anti-PCBP1 (ab168377, 1/10,000; Abcam) and loading control anti-GAPDH (ab8245, 1/10,000; Abcam) were used to incubate the membranes. This process still took one night. Then the membranes were incubated with the HRP-labeled secondary antibodies (Abcam) for 2 h at room temperature. The signals were detected on an ECL system (Amersham Pharmacia, Piscataway, New Jersey). GAPDH served as endogenous control.

### Northern Blot Analysis

RNA isolation from HUVEC cells treated with ox-LDL (50 μg/ml for 24 h) was conducted TRIZOL (Invitrogen). Probes of SHNG1 and GAPDH used for northern blotting were obtained using the Biotin RNA labeling mix (Roche). RNA samples were separated by electrophoresis and were transferred to the membranes (Amersham Pharmacia). Afterwards the membranes were incubated with the hydration buffer containing the probes. Finally, signals of SNHG1 and the internal control GAPDH were detected using the Chemiluminescent Nucleic Acid Detection Module (Thermo Scientific). This assay was repeated three times.

### RNA Pull-Down Assay

RNA pull-down assay was undertaken in treated HUVEC cells with ox-LDL (50μg/ml for 24 h). The utilization of T7 RNA polymerase was to add into a DNA fragment for taking the operation of PCR amplification. The full-length sequence was concluded in the DNA fragment. The restriction enzyme XhoI was utilized to linearize the plasmid DNA that was served as the product. The adoption of T7 RNA polymerase and Biotin RNA Labeling Mix (Roche) was utilized to take the inverse transcription on Biotin-labeled RNA. An RNeasy Mini Kit (Qiagen, MD, USA) was used to purify the product after RNase-free DNase I (Roche) was added to it, so as to create the RNA. In the end, the real-time PCR analysis used the RNA for operation.

### Subcellular Fractionation Assay

In accordance with the protocols of suppliers, Cytoplasmic and Nuclear RNA Purification Kit (Norgen, Thorold, ON, Canada) was used to separate cytoplasmic and nuclear fractions of ox-LDL-treated HUVEC cells (50 μg/ml for 24 h). Then qRT-PCR analysis was applied to detect the expressions of SNHG1 in different fractions, with GAPDH and U6 served as endogenous control.

### Statistical Analysis

Mean ± standard deviation (SD) was used to indicate all of the data obtained from more than three independent experiments. GraphPad Prism 6.5 (GraphPad Software, Inc., La Jolla, CA) was utilized to conduct statistical analyses. Comparisons between two groups were made by unpaired Student's t-test, whereas comparisons among more than two groups were made by one-way ANOVA followed by the Tukey and Donnet *post hoc* test. P < 0.05 was considered statistically significant.

## Results

### Establishment of ox-LDL-Induced HUVECs Model

**Figure 1:**
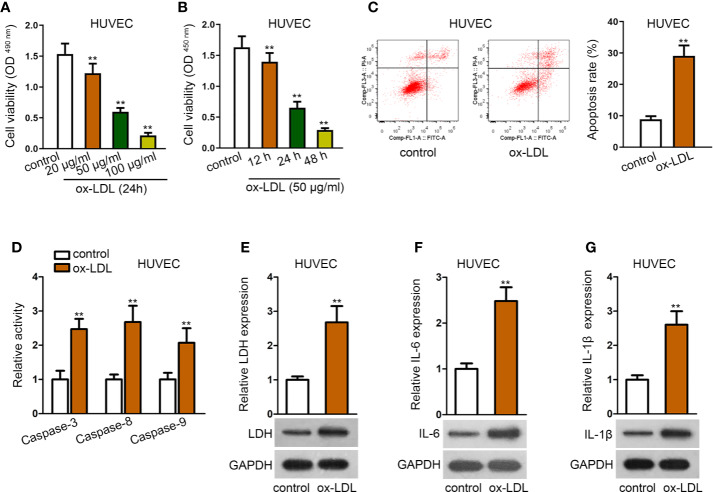
Establishment of ox-LDL induced HUVEC cell model. **(A, B)**. HUVECs were induced with ox-LDL at a concentration-independent or time-independent manner; then CCK-8 and MTT assay were applied to determine cell viability. ^**^P < 0.01 *versus* the control group. One-way ANOVA followed with the Donnet *post hoc* test. **(C)**. Cell apoptosis rate of HUVECs was evaluated by flow cytometry analysis. ^**^P < 0.01 *versus* the control group. Student t test. **(D)**. Protein activity of caspase-3/8/9 in HUVECs with or without ox-LDL treatment. ^**^P < 0.01 *versus* the control group. Student t test. **(E)**. LDH release was evaluated by a LDH Cytotoxicity Assay Kit; protein expression of LDH was evaluated by western blot in HUVECs with or without ox-LDL treatment. ^**^P < 0.01 *versus* the control group. Student t test. **(F, G)**. IL-6 **(F)**, and IL-1*β* **(G)** secretions were evaluated by Human High Sensitivity ELISA Kit; protein expression of IL-6 **(F)** and IL-1*β* **(G)** was detected by western blot in HUVECs with or without treatment of ox-LDL. ^**^P < 0.01 *versus* the control group. Student t test. Data obtained from more than three repeated experiments were shown as mean ± SD. **indicated P < 0.01 meant data were statistically significant.

The *in-vitro* cell model was firstly established *via* using ox-LDL to induce HUVECs. The viability of HUVECs was gradually reduced by ox-LDL treatment in a concentration-dependent or time-dependent manner, as shown in MTT and CCK-8 assays (**Figures 1A, B**). Also, cell apoptosis rate was significantly enhanced by ox-LDL treatment in flow cytometry analysis (**Figure 1C**). Relative activities of several apoptosis-related proteins, such as caspase-3, caspase-8, and caspase-9 were elevated in HUVECs after ox-LDL treatment (**Figure 1D**). To further explore the effect of ox-LDL on HUVECs, several factors related to inflammatory responses were evaluated. Expectedly, LDH release and protein expression of LDH were increased by ox-LDL treatment (**Figure 1E**). Moreover, we also detected the secretion and protein expression of IL-6 and IL-1*β* (pro-inflammatory cytokines). The results depicted that secretion and protein expression of IL-6 and IL-1*β* were significantly enhanced in ox-LDL-induced HUVECs than in the control group (**Figures 1F, G**). In general, ox-LDL induced cell apoptosis and inflammatory responses in HUVECs.

### Overexpression of SNHG1 Attenuated ox-LDL-Induced Cell Injury and Inflammatory Response in HUVECs

**Figure 2:**
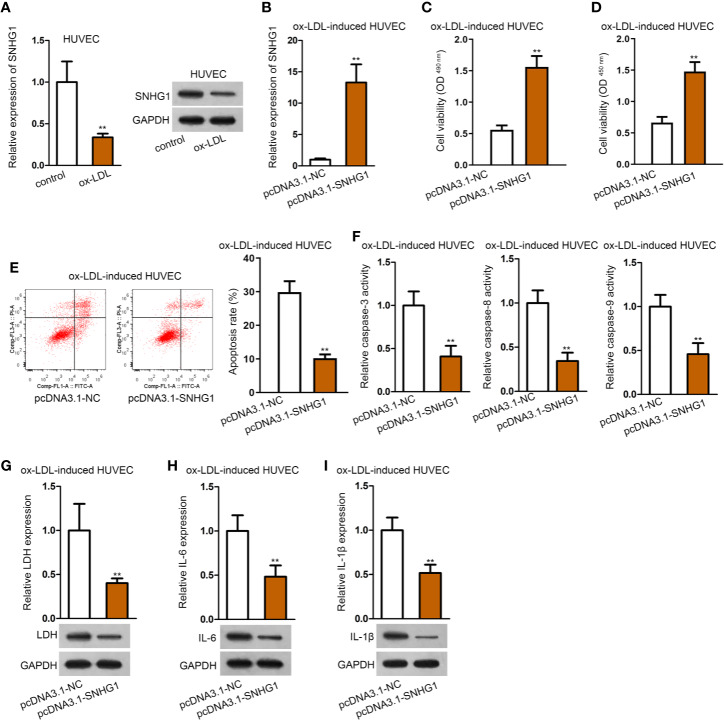
Overexpression of SNHG1 attenuated ox-LDL-induced cell injury and inflammatory response in HUVECs. **(A)** qRT-PCR and northern blot assay revealed SNHG1 expression. ^**^P < 0.01 *versus* the control group. Student t test. **(B)** Overexpression efficiency of SNHG1 was validated by qRT-PCR. ^**^P < 0.01 *versus* the pcDNA3.1-NC group. Student t test. **(C, D)** CCK-8 and MTT assay revealed cell viability of HUVECs transfected with pcDNA3.1-SNHG1. ^**^P < 0.01 *versus* the pcDNA3.1-NC group. Student t test. **(E)** Cell apoptosis rate in HUVECs transfected with pcDNA3.1-SNHG1 was evaluated in flow cytometry analysis. ^**^P < 0.01 *versus* the pcDNA3.1-NC group. Student t test. **(F)** Caspase-3/8/9 protein activity was detected in HUVECs transfected with pcDNA3.1-SNHG1. ^**^P < 0.01 *versus* the pcDNA3.1-NC group. Student t test. **(G)** LDH release was evaluated by a LDH Cytotoxicity Assay Kit; protein expression of LDH was evaluated by western blot in HUVECs transfected with pcDNA3.1-SNHG1. ^**^P < 0.01 *versus* the pcDNA3.1-NC group. Student t test. **(H, I)**. IL-6 **(H)**, and IL-1*β* **(I)** secretion were evaluated by Human High Sensitivity ELISA Kit; protein expression of IL-6 **(H)** and IL-1*β* **(I)** was detected by western blot in HUVECs transfected with pcDNA3.1-SNHG1. ^**^P < 0.01 *versus* the pcDNA3.1-NC group. Student t test. Data obtained from more than three repeated experiments were shown as mean ± SD. ** indicated P < 0.01 meant data were statistically significant.

Then, we observed that the expression of SNHG1 was notably under-expressed in ox-LDL-induced HUVECs compared with the control group (**Figure 2A**). Next, pcDNA3.1-SNHG1 was transfected into ox-LDL-induced HUVECs. The overexpression efficiency of SNHG1 was verified by qRT-PCR analysis (**Figure 2B**). Data of CCK-8 and MTT assays revealed that SNHG1 up-regulation alleviated the suppressive effects of ox-LDL on the viability of HUVECs (**Figures 2C, D**). Also, flow cytometry revealed that over-expression of SNHG1 notably reduced cell apoptosis rate in ox-LDL-induced HUVECs (**Figure 2E**). Activities of apoptosis-related proteins were decreased by overexpression of SNHG1 (**Figure 2F**). Next, LDH release and protein expression of LDH were reduced by up-regulated SNHG1 in ox-LDL-induced HUVECs (**Figure 2G**). Further, secretion of IL-6 and IL-1*β* as well as their protein expression was apparently suppressed by treatment of pcDNA3.1-SNHG1 (**Figures 2H, I**). Thus, we concluded that SNHG1 played a vital role in the dysfunction of HUVECs. Overexpression of SNHG1 attenuated ox-LDL-induced injury and inflammatory response in HUVECs.

### SNHG1 Acted as miR-556-5p Sponge to Up-Regulate GNAI2

**Figure 3:**
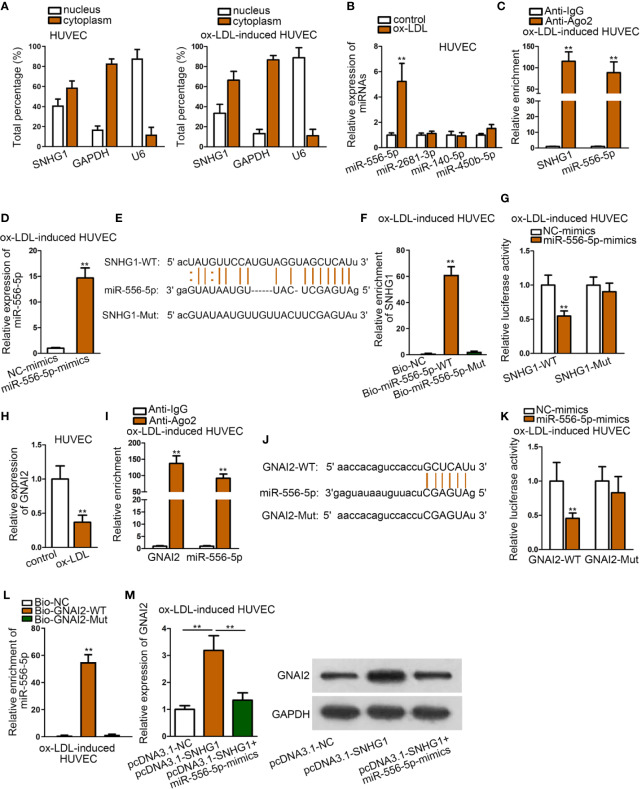
SNHG1 Sponged miR-556-5p to Up-regulate GNAI2. **(A)** Subcellular fraction assay revealed the subcellular location of SNHG1 in basal HUVECs and ox-LDL-induced HUVECs. **(B)** qRT-PCR was applied to examine expression of four candidate miRNAs in ox-LDL-induced HUVECs. ^**^P < 0.01 *versus* the control group. Student t test. **(C)**. RIP assay was applied to detect relative enrichment of SNHG1 and miR-556-5p in Ago2 and IgG group. ^**^P < 0.01 *versus* the anti-IgG group. Student t test. **(D)** qRT-PCR verified the overexpression efficiency of miR-556-5p. ^**^P < 0.01 *versus* the NC-mimics group. Student t test. **(E)** Binding sequences of wild type/mutant SNHG1 and miR-556-5p were predicted from “starBase”. **(F)** RNA pull-down assay examined relative enrichment of SNHG1 pulled down by biotin-labeled wild type/mutant miR-556-5p. ^**^P < 0.01 *versus* the Bio-NC group. Student t test. **(G)** Luciferase activity of wild and mutant SNHG1 by overexpression of miR-556-5p was examined in luciferase reporter assay. ^**^P < 0.01 *versus* the NC-mimics group. Student t test. **(H)** qRT-PCR examined GNAI2 expression in HUVECs. ^**^P < 0.01 *versus* the control group. Student t test. **(I)** RIP assay detected relative enrichment of GNAI2 and miR-556-5p in Ago2 and IgG group. ^**^P < 0.01 *versus* the anti-IgG group. Student t test. **(J)** Binding sites of wild type/mutant GNAI2 and miR-556-5p were predicted from “starBase” database. **(K)** Luciferase activity of wild and mutant GNAI2 by overexpression of miR-556-5p was examined in luciferase reporter assay. ^**^P < 0.01 *versus* the NC-mimics group. Student t test. **(L)** RNA pull-down assay examined relative enrichment of GNAI2 pulled down by biotin-labeled wild type/mutant miR-556-5p. ^**^P < 0.01 *versus* the Bio-NC and Bio-GNAI2-mut group. Student t test. **(M)** qRT-PCR detected GNAI2 expression in HUVECs by indicated transfections. ^**^P < 0.01 of the 2^nd^ group *versus* the 1^st^ group and 3^rd^ group *versus* 2^nd^ group. One-way ANOVA followed with the Tukey *post hoc* test. Data obtained from more than three repeated experiments were shown as mean ± SD. ** indicated P < 0.01 meant data were statistically significant.

We firstly detected the cellular location of SNHG1 by subcellular fraction assay. It was revealed that SNHG1 was mainly located in the cytoplasm of HUVECs, regardless of ox-LDL treatment (**Figure 3A**). Since the ceRNA mechanism is a typical post-transcriptional network, we hypothesized that SNHG1 functioned as a ceRNA in ox-LDL-induced HUVECs. Using “starBase” algorithm (Li et al., 2013), four miRNAs were identified to be the downstream molecules of SNHG1 (CLIP data: strict stringency; degradome data: high stringency; pan-cancer: at least two cancer types). Among the four miRNAs, only miR-556-5p was up-regulated in ox-LDL-induced HUVECs, while the expression of the other three miRNAs showed no significant changes (**Figure 3B**). The following RIP assay verified that SNHG1 and miR-556-5p were significantly enriched in Ago2-precipitated RISCs (RNA induced silence complexes) (**Figure 3C**). Next, we enhanced the expression of miR-556-5p in ox-LDL-induced HUVECs for the following assays. The overexpression efficiency of miR-556-5p was validated by qRT-PCR analysis (**Figure 3D**). Then, we obtained the binding sequences of miR-556-5p and SNHG1 from starBase tool and mutated the binding sequences of SNHG1. **Figure 3E** revealed the binding sites of wild type/mutant SNHG1 and miR-556-5p. In the RNA pull-down assay, SNHG1 was apparently pulled down by biotin-labeled wild miR-556-5p rather than biotin-labeled mutant group (**Figure 3F**). According to the result of the luciferase reporter assay, the luciferase activity of wild type SNHG1 was evidently decreased by miR-556-5p overexpression, while luciferase activity of mutant SNHG1 was not impacted under the same conditions (**Figure 3G**). All these assays indicated that SNHG1 sponged miR-556-5p at predicted binding sites. Next, we chose G protein subunit alpha i2 (GNAI2) as the candidate target gene of miR-556-5p by means of “starBase” (CLIP data: strict stringency; degradome data: high stringency; pan-cancer: at least six cancer types). GNAI2 was down-regulated in ox-LDL-induced HUVECs (**Figure 3H**). MiR-556-5p and GNAI2 were abundantly enriched in anti-Ago2 precipitated RISCs (**Figure 3I**). Then, we mutated the site of GNAI2 where harbored the binding sites with miR-556-5p, and **Figure 3J** revealed the binding sites of wild type/mutant GNAI2 and miR-556-5p. The following RNA pull-down assay and luciferase reporter assay indicated that miR-556-5p targeted GNAI2 at predicted sites (**Figures 3K, L**). Finally, we figured out that SNHG1 up-regulation enhanced the expression of GNAI2, while cotransfection of miR-556-5p mimics reduced GNAI2 expression (**Figure 3M**). To sum up, SNHG1 sponged miR-556-5p to up-regulate GNAI2 expression.

### SNHG1 Regulated ox-LDL-Induced Cell Injury and Inflammatory Response *via* Down-Regulating miR-556-5p and Up-Regulating GNAI2

**Figure 4:**
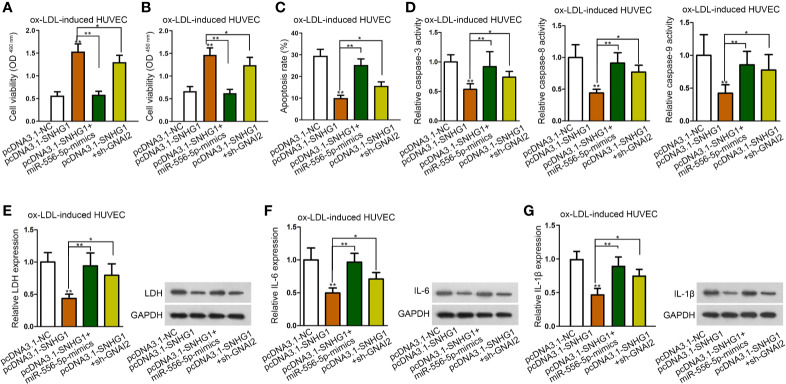
SNHG1 regulated ox-LDL-induced cell injury and inflammatory response *via* down-regulating miR-556-5p and up-regulating GNAI2. **(A, B)** CCK-8 and MTT assay respectively determined cell viability of ox-LDL-induced HUVECs under indicated transfections. One-way ANOVA followed with the Tukey *post hoc* test. **(C)**–**(G)**. Cell apoptosis rate **(C)**, caspase-3/8/9 activity **(D)**, LDH release and protein expression **(E)**, IL-6 secretion and protein expression **(F)**, IL-1*β* secretion and protein expression **(G)** in ox-LDL-induced HUVECs in four different groups were determined. Four groups in the experiments in this figure: pcDNA3.1-NC, pcDNA3.1-SNHG1 (^**^P < 0.01 *versus* the 1^st^ group), pcDNA3.1-SNHG1+miR-556-5p-mimics (^**^P < 0.01 *versus* the 2^nd^ group), pcDNA3.1-SNHG1+sh-GNAI2 (^*^P < 0.05 *versus* the 2^nd^ group). One-way ANOVA followed with the Tukey *post hoc* test. Data obtained from more than three repeated experiments were shown as mean ± SD. * indicated P < 0.05 and ** indicated P < 0.01 meant data were statistically significant.

To further explore whether miR-556-5p and GNAI2 were required for SNHG1-mediated dysfunction of HUVECs, a range of rescue assays were conducted. According to the results of CCK-8 and MTT assay, up-regulated miR-556-5p and silenced GNAI2 counteracted the promoting effects of SNHG1 on cell viability (**Figures 4A, B**). Moreover, the suppressive effects of SNHG1 on cell apoptosis were restored by miR-556-5p-mimics and sh-GNAI2 (**Figures 4C, D**). Besides, miR-556-5p overexpression and GNAI2 depletion evidently restored the suppressive effects of SNHG1 on the inflammatory response in HUVECs (**Figures 4E–G**). Interestingly, GNAI2 and miR-556-5p partially and completely rescued the effects of SNHG1 overexpression. Thus, we hypothesized that there was another mediator downstream SNHG1/miR-556-5p axis in ox-LDL-induced HUVECs.

### MiR-556-5p Targeted Both GNAI2 and PCBP1 in ox-LDL-Induced HUVECs

**Figure 5:**
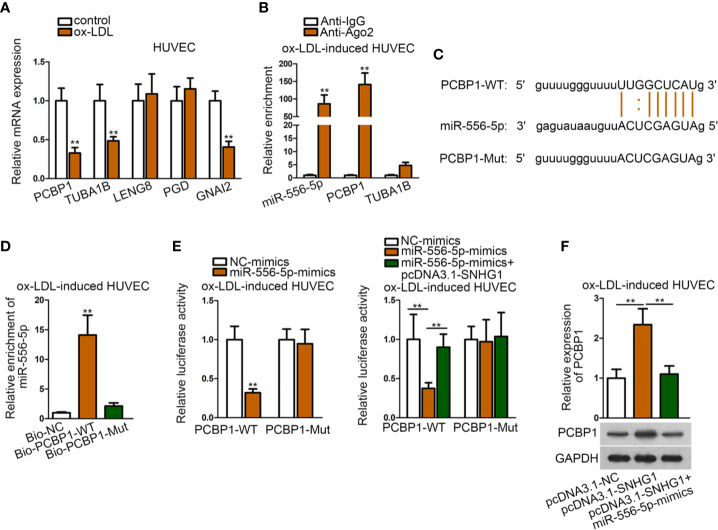
MiR-556-5p targeted both GNAI2 and PCBP1 in ox-LDL-induced HUVECs. **(A)** qRT-PCR examined expression of five target genes in ox-LDL-induced HUVECs. ^**^P < 0.01 *versus* the control group. Student t test. **(B)** RIP assay detected relative enrichment of miR-556-5p, PCBP1, TUBA1B in Ago2 and IgG group. ^**^P < 0.01 *versus* the anti-IgG group. Student t test. **(C)** Binding sequences of wild type/mutant PCBP1 and miR-556-5p were predicted from “starBase”. **(D)** RNA pull-down assay examined relative enrichment of PCBP1 pulled down by wild type miR-556-5p at predicted sites. ^**^P < 0.01 *versus* the Bio-NC group. Student t test. **(E)** Luciferase reporter assay detected luciferase activity of PCBP1 in HUVECs by indicated transfections. The left panel: ^**^P < 0.01 *versus* the NC-mimics group; the right panel: ^**^P < 0.01 *versus* the 1^st^ and 3^rd^ groups. **(F)** qRT-PCR and western blot analysis revealed PCBP1 expression in HUVECs by indicated transfections. ^**^P < 0.01 *versus* the 1^st^ and 3^rd^ groups. One-way ANOVA followed by Tukey *post hoc* test. Data obtained from more than three repeated experiments were shown as mean ± SD. ** indicated P < 0.01 meant data were statistically significant.

Based on the above results, we sought to search for another target of miR-556-5p by “starBase” screened by different criteria (CLIP data: strict stringency; degradome data: high stringency). Accordingly, five mRNAs were identified. Among them, PCBP1 (poly(rC) binding protein 1), TUBA1B (tubulin alpha 1b), and GNAI2 were lowly-expressed in ox-LDL-induced HUVECs (**Figure 5A**). Then, we detected relative enrichment of miR-556-5p, PCBP1, and TUBA1B precipitated by IgG or Ago2 in RIP assay. The result indicated that only PCBP1 and miR-556-5p were precipitated by Ago2, indicating that PCBP1 could interact with miR-556-5p (**Figure 5B**). The binding sites of PCBP1 and miR-556-5p were predicted from “starBase”, and we mutated the binding sequences of PCBP1 (**Figure 5C**). The following RNA pull-down assay and luciferase reporter assay revealed that the predicted binding sites were responsible for the interaction of miR-556-5p and PCBP1 (**Figures 5D, E**). It was also revealed in the luciferase reporter assay that cotransfection of pcDNA3.1-SNHG1 enhanced luciferase activity of PCBP1, which was previously decreased by miR-556-5p mimics (**Figure 5E**). Further, we observed that miR-556-5p overexpression could rescue the positive effects of up-regulated SNHG1 on PCBP1 expression (**Figure 5F**). Thus, we concluded that SNHG1 up-regulate PCBP1 expression through acting as the sponge of miR-556-5p.

### SNHG1 Inhibited ox-LDL-Induced Cell Injury and Inflammatory Response *via* Up-Regulating GNAI2 and PCBP1

**Figure 6:**
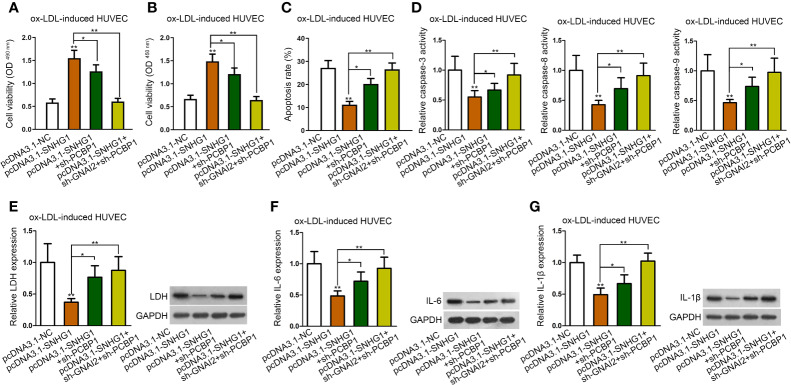
SNHG1 inhibited ox-LDL-induced cell injury and inflammatory response *via* up-regulating GNAI2 and PCBP1. **(A, B)** CCK-8 and MTT assay determined cell viability of indicated ox-LDL-induced cells. **(C, G)**. Cell apoptosis rate **(C)**, caspase-3/8/9 activity **(D)**, LDH release and protein expression **(E)**, IL-6 secretion and protein expression **(F)**, IL-1*β* secretion and protein expression **(G)** in ox-LDL-induced HUVECs in indicated groups. Four groups in the experiments: pcDNA3.1-NC, pcDNA3.1-SNHG1 (^**^P < 0.01 *versus* the 1^st^ group), pcDNA3.1-SNHG1+sh-PCBP1 (^*^P < 0.05 *versus* the 2^nd^ group), pcDNA3.1-SNHG1+sh-GNAI2/PCBP1 (^**^P < 0.01 *versus* the 2^nd^ group). One-way ANOVA followed by Tukey *post hoc* test. Data obtained from more than three repeated experiments were shown as mean ± SD. * indicated P < 0.05 and ** indicated P < 0.01 meant data were statistically significant.

To verify whether both PCBP1 and GNAI2 were required for SNHG1-mediated cell injury and inflammatory response of HUVECs, the final rescue assays were conducted. As was shown in CCK-8 and MTT assay, inhibition of PCBP1 alone could partly, whereas cosuppression of GNAI2 and PCBP1 completely counteracted the promoting effects of up-regulated SNHG1 on cell viability (**Figures 6A, B**). Also, up-regulated SNHG1-reduced cell apoptosis was partially restored by PCBP1 knockdown but enhanced to normal by depletion of both PCBP1 and GNAI2 (**Figures 6C, D**). Next, SNHG1-mediated reduction on HUVEC inflammatory response was reversed by PCBP1 inhibition in part but rescued absolutely by coinhibition of PCBP1 and GNAI2 (**Figures 6E–G**). Based on these results, we concluded that SNHG1 attenuated ox-LDL-induced cell injury and inflammatory response *via* up-regulating GNAI2 and PCBP1 in HUVECs.

## Discussion

Atherosclerosis is a common cardiovascular disease and has caused a great threat to human health. EC dysfunction is an important marker for atherosclerosis. Previous studies have revealed the regulatory impact of various lncRNAs on the dysfunction of ECs. Present study was designed to probe into the biological role of SNHG1 which has been implicated in atherosclerosis-caused cerebral infraction and could regulate cardiovascular disorders. We used ox-LDL to stimulate cell injury and inflammatory response of HUVECs. Then, we observed that SNHG1 up-regulation alleviated ox-LDL-stimulated cell apoptosis, injury, or inflammatory response in HUVECs.

SNHG1 has reported to function as the ceRNA in various diseases. For instance, SNHG1 acts as a ceRNA of miR-137 in neuronal cells to aggravate Aβ25-35-inudced neuronal injury *via* up-regulating KREMEN1 (Wang et al., 2019). Overexpression of SNHG1 facilitates the proliferation *via* miR-488-5p/NUP205 pathway in acute myeloid leukemia cells (Bao et al., 2019). SNHG1 aggravates malignancy of glioma cells through functioning as a sponge of miR-154-5p/miR-376b-3p to up-regulate FOXP2 (Li et al., 2019). SNHG1 competitively binds to miR-194 to relieve the inhibitory effect of miR-194 on PHLDA1 expression, thus promoting glioma progression (Liu et al., 2019).

By usage of bioinformatics analysis and mechanism assays, we unveiled that miR-556-5p was the downstream molecule of SNHG1. SNHG1 competed with GNAI2 to bind miR-556-5p. GNAI family plays a vital role in cardiovascular or cardiovascular-related disorders. RXFP1 is activated by Relaxin-2 and induces vascular relaxation *via* a Gn*α*i2-protein/PI3K*ß*/*γ*/Nitric oxide-Coupled pathway (Lian et al., 2018). The absence of GNAI2 significantly increases the infarct size in the heart, indicating the protective role of GNAI2 in cardiac ischemia reperfusion injury (Kohler et al., 2014). Next, the functional rescue assays revealed that overexpression of SNHG1 inhibited ox-LDL-induced cell injury and inflammatory response *via* down-regulating miR-556-5p and up-regulating GNAI2. More importantly, the rescue effects of GNAI2 were not as significant as the rescue effects of miR-556-5p, which indicated that SNHG1 sponged miR-556-5p to regulate another target of miR-556-5p. Thereafter, we identified PCBP1 as another target of miR-556-5p. PCBP1 belongs to the Poly(C)-binding proteins (PCBPs) family which interacts in a sequence-specific fashion with single-stranded poly(C). Among PCBP family, PCBP2 functions as an anti-hypertrophic factor (Zhang et al., 2015). A previous study has reported that PCBP1 protein mediates pro-inflammatory cytokine production *via* stabilizing Csf2 and Il2 (Wang et al., 2018). Also, PCBP1 plays a as possible regulator in neurodegenerative disease (Geuens et al., 2017). On this basis, present study uncovered that SNHG1 attenuated atherosclerosis *via* up-regulating GNAI2 and PCBP1.

Previously, Liang S et al. elucidated that SNHG1 serves as a ceRNA to alleviate hypoxia-reoxygenation (H/R)-induced HUVEC injury through the HIF-1*α*/VEGF pathway (Liang et al., 2020). Compared to that study, we focused on molecular mechanism of ox-LDL-induced HUVEC apoptosis, injury and inflammatory response, while Liang S et al. focused on H/R-induced HUVEC proliferation, migration and angiogenesis. Besides, our study uncovered the novel ceRNA axis of SNHG1/miR-556-5p/GNAI2-PCBP1, while Liang S et al. revealed the SNHG1/miR-140-3p/HIF-1*α*/VEGF axis. Moreover, Wang Z et al. uncovered that SNHG1 facilitates the angiogenesis of brain microvascular endothelial cells (BMECs) under oxygen-glucose deprivation/reoxygenation (OGD/R) condition (Wang et al., 2018). Yang X and Zi XH demonstrated that SNHG1 attenuates OGD induced injury in BMEC through miR-338/HIF-1*α* axis (Yang and Zi, 2019). Wang Z et al. focused on angiogenesis of BMECs under OGD/R condition and revealed miR-199a as the down-stream molecule of SNHG1. Yang X and Zi XH focused on BMEC apoptosis under OGD and revealed miR-338/HIF-1*α* as the down-stream axis of SNHG1. Compared to previous studies, present study focused on cell apoptosis, injury and inflammatory response of ox-LDL-induced HUVECs, and revealed miR-556-5p/GNAI2-PCBP1 as the downstream pathway of SNHG1.

In conclusion, this research unveiled a novel ceRNA axis of SNHG1/miR-556-5p/GNAI2-PCBP1 in ox-LDL-induced HUVECs. We innovatively revealed that SNHG1 inhibited ox-LDL-induced inflammatory response and apoptosis of HUVECs *via* up-regulating GNAI2 and PCBP1 in a miR-556-5p dependent way. Lack of animal study is the limitation of this study. We will conduct *in vivo* study to support the result of our current study in the future. Moreover, we will also explore the upstream molecular mechanism of SNHG1 in the cell model in our future study.

## Data Availability Statement

The raw data supporting the conclusions of this article will be made available by the authors, without undue reservation, to any qualified researcher.

## Author Contributions

YL: Conception and design. JX: Development of methodology. YZ: Acquisition of data. WC: Analysis and interpretation of data. FZ: Writing and revising the manuscript. CL: Administrative, technical and material support. ZW: Study supervision.

## Funding

Youth Science Foundation Project: MiR-34a optimizes bone marrow mesenchymal stem cells to treat diabetic cardiomyopathy by regulating autophagy.

## Conflict of Interest

The authors declare that the research was conducted in the absence of any commercial or financial relationships that could be construed as a potential conflict of interest.

## Supplementary Material

Supplementary Table 1 Sequences of PCR primers.
