# Supplementary material for: SNHG1 Inhibits ox-LDL-Induced Inflammatory Response and Apoptosis of HUVECs via Up-Regulating GNAI2 and PCBP1
Source: Front Pharmacol. 2020 May 27;11:703. doi: 10.3389/fphar.2020.00703 (PMC7266976; doi:10.3389/fphar.2020.00703)
Supplement: Supplementary Table 1 — Sequences of PCR primers. [file Table_1.docx]

**Relative sequences of PCR primers (5’-3’)**

| SNHG1 | F: | CCCCTGTTTTAGCCTGGGAA |
| --- | --- | --- |
|  | R: | ACCTTTGAGCCAAGCAGGTT |
| PCBP1 | F: | AAGAAAGGGGAGTCGGTTAAGA |
|  | R: | GCCGGTCAGAGTGATGATTCTC |
| TUBA1B | F: | ACCTTAACCGCCTTATTAGCCA |
|  | R: | ACATTCAGGGCTCCATCAAATC |
| LENG8 | F: | ACGTAGCACAGATTGGTCTTCT |
|  | R: | CTCCGGGTTCTCGTGCATC |
| PGD | F: | ATGGCCCAAGCTGACATCG |
|  | R: | AAAGCCGTGGTCATTCATGTT |
| GNAI2 | F: | TACCGGGCGGTTGTCTACA |
|  | R: | GGGTCGGCAAAGTCGATCTG |
| GAPDH | F: | CTGGGCTACACTGAGCACC |
|  | R: | AAGTGGTCGTTGAGGGCAATG |
| miR-556-5p | F: | GGCAGGGATGAGCTCATTGTA |
|  | R: | CTCAACTGGTGTCGTGGA |
| miR-2681-3p | F: | GGCAGGTATCATGGAGTTGGTA |
|  | R: | CTCAACTGGTGTCGTGGA |
| miR-140-5p | F: | GCCGAGCAGTGGTTTTACCCT |
|  | R: | CTCAACTGGTGTCGTGGA |
| miR-450b-5p | F: | TCGGCAGGTTTTGCAATATGTTC |
|  | R: | CTCAACTGGTGTCGTGGA |
| U6 | F: | GCAGACCGTTCGTCAACCTA |
|  | R: | AATTCTGTTTGCGGTGCGTC |

F meant forward primer and R meant reverse primer.
